# Larvicidal Activity of Essential Oil of *Syzygium aromaticum* (Clove) in Comparison with Its Major Constituent, Eugenol, against *Anopheles stephensi*

**Published:** 2018-12-25

**Authors:** Mahmoud Osanloo, Mohammad Mehdi Sedaghat, Fariba Esmaeili, Amir Amani

**Affiliations:** 1 Department of Medical Nanotechnology, School of Advanced Technologies in Medicine, Tehran University of Medical Sciences, Tehran, Iran; 2 Students’ Scientific Research Center, Tehran University of Medical Sciences, Tehran, Iran; 3 Department of Medical Entomology and Vector Control, School of Public Health, Tehran University of Medical Sciences, Tehran, Iran; 4 Natural Products and Medicinal Plants Research Center, North Khorasan University of Medical Sciences, Bojnurd, Iran

**Keywords:** Larvicidal activity, HPLC, *Syzigium aromaticum*, Eugenol, Essential oil, *Anopheles stephensi*

## Abstract

**Background:**

In this study, larvicidal activity of clove essential oil (EO), as a green and relatively potent larvicide, was compared with its main constituent, Eugenol, against *Anopheles stephensi*.

**Methods:**

High-performance liquid chromatography (HPLC) was used to determine the amount of eugenol, major constituent of clove EO. In addition, larvicidal activity of clove EO and eugenol was evaluated against *An. stephensi.*

**Results:**

The amount of eugenol in clove EO was determined as 67% using HPLC analysis. LC_50_ and LC_90_ of clove EO (57.49 and 93.14ppm, respectively) were significantly lower than those of eugenol (86.96 and 128.18 ppm, respectively).

**Conclusion:**

EO showed more effective than its major component. Considering the lower cost of the essential oil and lower risk in occurrence of resistance in larvae, use of clove EO is preferred as larvicide in comparison with eugenol, against *An. stephensi.*

## Introduction

More than 17% of all infectious diseases around the world are vector-borne diseases, such as dengue fever, yellow fever, and malaria (1). The number of death for such diseases is more than 1 million annually e.g. malaria caused 429000 death just in 2015 (1, 2). In order to control malaria, WHO recommends control of larva which now used in 55 countries (2). Unfortunately, due to frequent use of synthetic larvicides (such as Temephos), not only environmental pollution have appeared, but also many cases of resistance has occurred in mosquitoes around the world (3–6). Essential oils (EOs) have been suggested as alternative sources for insect control as repellents, insecticides or larvicides and they offer advantages such as biodegradability and negligible effects on non-target specious and environment (7, 8).

Recently, comparisons of larvicidal activity of EOs with their major components have been reported (9, 10). However, there is no conclusion so fare, EOs are better or their major constituent(s) in terms of larvicidal activity.

*Syzygium aromaticum* (Clove) belongs to the Myrtaceae family which considers as an important medicinal plant with wide range of biological activities such as anti-bacterial or anti-oxidant activities (11, 12). Clove EO has also shown larvicidal activity against field collected larva of *Ae. Aegypti* with LC_50_ of 92.56 and 62.3ppm in two different reports (13, 14). Eugenol, as the major constituent of clove EO, has also indicated larvicidal activity against laboratory reared and field collected of *Ae. aegypti* (LC_50_: 33 and 93.3ppm, respectively) (14, 15). Its larvicidal activity against other population of mosquito has been documented, with LC_50_ of 25.4, 28.14 and 30.8ppm against *An. subpictus*, *Ae. albopictus* and *Cx. tritaeniorhynchus* (16).

*Anopheles stephensi* is an important malaria vector with wide distribution in the Arabian Peninsula, the Indian subcontinent, Afghanistan and Iran (17–19). In this research, for the first time, larvicidal activity of clove EO and eugenol against *An. stephensi* was evaluated and compared.

## Materials and Methods

### Materials

High-performance liquid chromatography (HPLC) grade methanol, pure eugenol (99%) and Ethanol were supplied by Merck (Germany). Clove EO was purchased from Green Plants of Life Co (Iran).

### Determining of eugenol contents in clove EO oil by HPLC

HPLC analysis was used to determine the amount of eugenol in clove EO. The apparatus consisted of a 30cm× 3.9mm reverse phase C18 column (Waters, Milford, USA), a pressure less injection pump (Model L-6200, Hitachi, Japan) to drive solvent and loading of samples, a UV visible detector (Model L-4000, at 280nm, Hitachi, Japan) for detection, a chromato-integrator (Model D-2500, Hitachi, Japan) for analyses. A mixture of methanol and distilled water was used as mobile phase with flow-rate of 1mL/min. Analytical procedure was started with dissolving 50μL of eugenol or 0.5mL of clove EO in 10mL of methanol, then, 20μL of this solution was injected into system at a flow-rate of 0.7mL/min. The optimum mobile phase with a methanol: water ratio of 80:20 was used for elution. By comparing peak areas of eugenol with solution of clove EO, its amount in clove EO was determined.

### Evaluation of larvicidal activity

Third and fourth instar larvae of *An. stephensi* were used, they obtained from insectarium of Tehran University of Medical Sciences. They reared in special condition: 28± 2 °C, 12:12 dark and light periods and relative humidity of 65±5%. Larvicidal bioassay was performed in line with WHO recommendation test in lab, with some modifications (20). Standard solutions were prepared by dissolving in ethanol at appropriate concentrations (i.e. eugenol 60 μL/mL and clove EO 30μL/mL). By adding 1mL from each sample (0.5%v/v) to cups containing 199mL of no chlorine water, desired concentrations of samples were prepared. Using separated nets, 25 larvae of *An. stephensi* were added slowly to all containers. Dead and moribund larvae (unable to respond to stimulating agent) were counted after 24h of exposing in all cups. Larvicidal bioassays were performed in 16 repetitions at 4 different replicates at concentrations (ppm) of 12.5, 25, 50, 75, 100, 150 for clove EO and 12.5, 25, 50, 100, 150, 200 and 300 for eugenol. In each replicate, two control groups were considered having ethanol (0.5%v/v) with similar treatments. Lethal concentrations of each sample (i.e. LC_50_ and LC_90_), were determined using a probit regression model in SPSS ver. 19 (Chicago, IL, USA).

### Comparison of larvicidal activity of clove EO with eugenol

Evaluation of overlaps between confidence intervals (CI) of two groups is an easy and common approach to compare various LC values. If no overlap is observed, the difference is considered as significant (21). LC_50_ and LC_90_ of clove EO and eugenol were calculated and compared using independent sample test by SPSS.

## Results

### Determining content of eugenol in clove EO

**Fig. 1.**
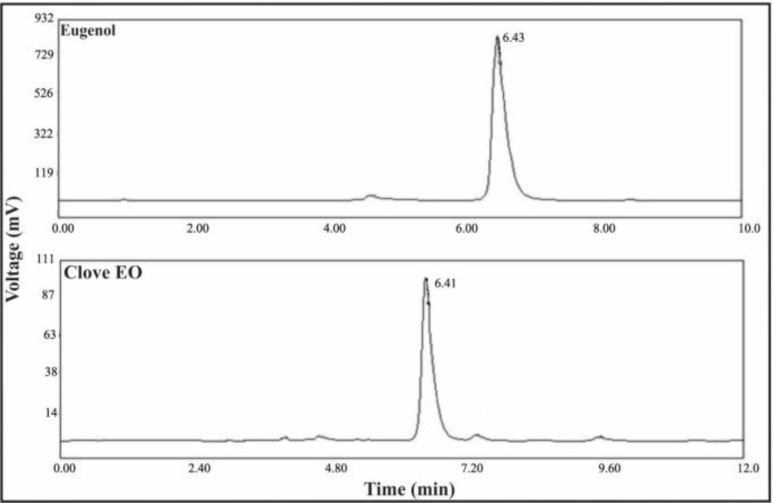
HPLC profile of solution of eugenol and clove EO, related peak for eugenol appeared at retention time of 6.43 and 6.41min, respectively

Results of HPLC analysis of eugenol and clove EO samples are shown in Fig. 1. Comparing the graphs, the peak related to eugenol is observed at retention time of 6.43 and 6.41min, for eugenol and clove EO, respectively. By comparing peak areas of eugenol and clove EO, percentage of eugenol in clove EO was calculated as 67%, considered relatively high.

### Evaluation of larvicidal activity of clove EO and eugenol

**Fig. 2.**
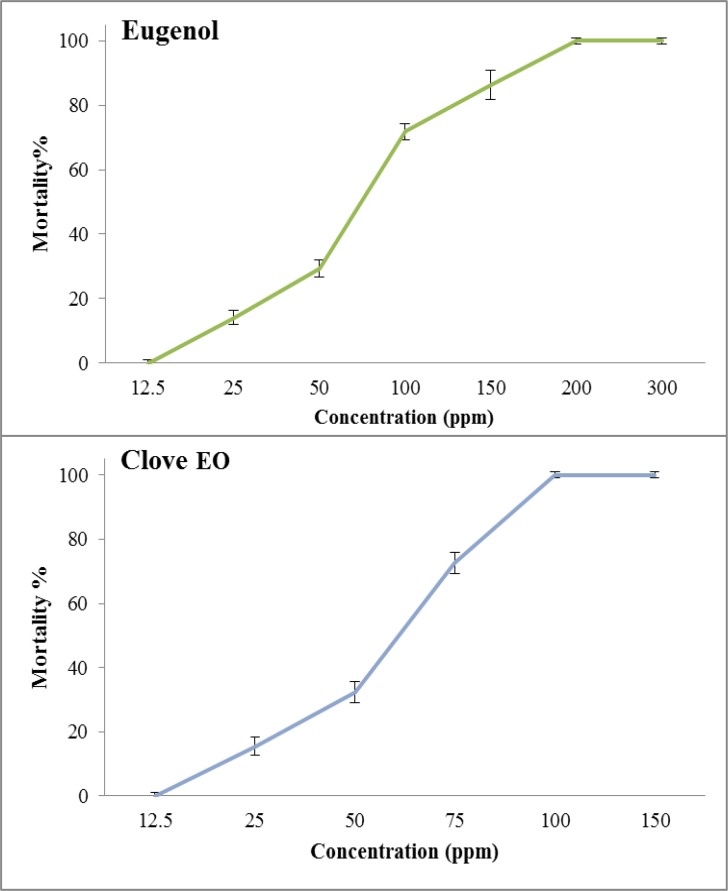
Larvicidal activity of clove EO and eugenol against *Anopheles stephensi*

Results of larvicidal activities of clove EO and eugenol against *An. stephensi* are shown in Fig. 2. Calculated LC_50_ and LC_90_ values were 57.49 and 86.96ppm for clove EO and 93.14 and 158.2ppm for eugenol, respectively (Table 1).

**Table 1.** Calculated parameters by probit analysis

**Specimen**	**A**	**B±SE**	**LC_50_ (ppm) CI: (LCL-UCL)**	**LC_90_ (ppm) CI: (LCL–UCL)**	**χ^2^ (df)**	**Sig**
**Clove EO**	−2.50	0.04±0.002	57.49 (43.28–74.24)	86.96 (71.19–128.18)	47.96 (3)	0.15 > sig*
**Eugenol**	−1.83	0.02±0.001	93.14 (75.60–113.33)	158.2 (133.85–201.20)	39.13 (4)	0.15 > sig*

A= intercept; B±SE= slope and standard error of the line; CI= confidence interval (0.05), UCL= Upper Confidence Limit, LCL= Lower Confidence Limit, χ^2^ (df)= Chi 2 and degree of freedom.

* Since the significance level is less than 0.15, a heterogeneity factor is used in the calculation of confidence limits.

**Fig. 3.**
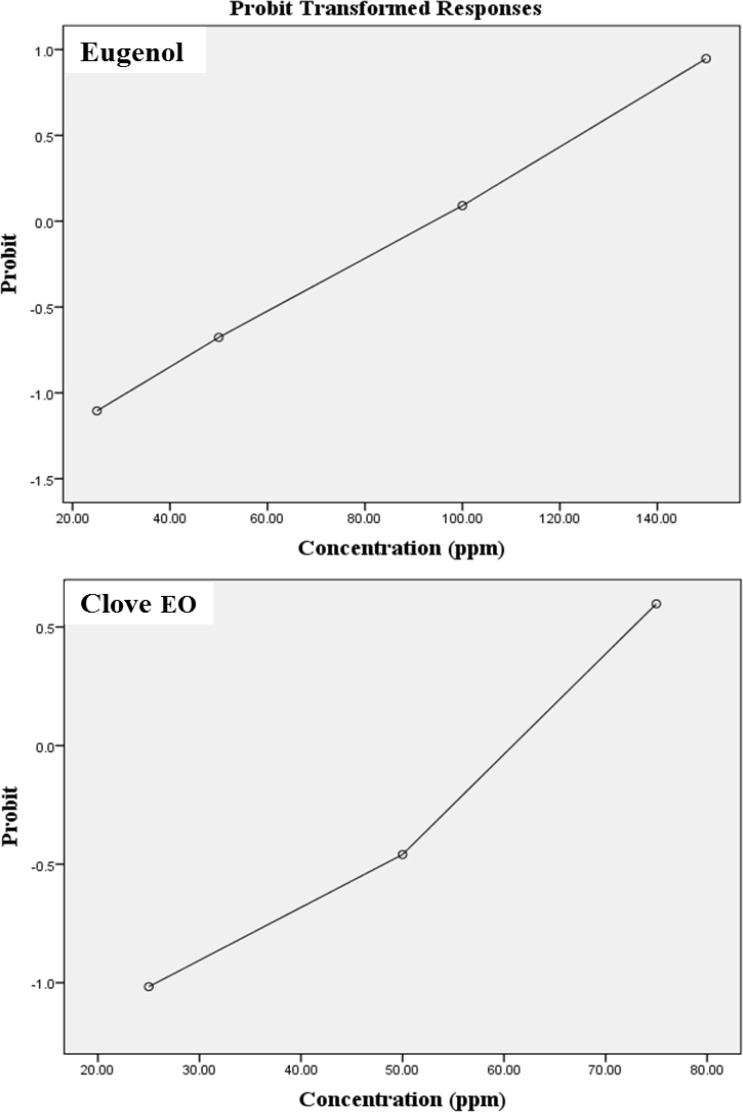
Probit regression line of clove EO and eugenol

Larvicidal activity in both samples (i.e. clove EO and eugenol) appeared at 25ppm and enhanced with increasing the concentration of those. Nevertheless, perfect larvicidal activities were achieved at 100 and 200ppm for clove EO and eugenol, respectively. Furthermore, no overlap in CI of LC_50_ and LC_90_ for clove EO and eugenol are observed, thus, larvicidal activity of clove EO is significantly better than eugenol (Table 1). Moreover, Probit regression line of the both clove EO and eugenol are illustrated in Fig. 3.

## Discussion

### Determining content of eugenol in clove EO

In this study, amount of eugenol in clove EO was found as 67%, which is comparable with other reports. Reviewing other reports, content of eugenol has been reported in values more or less similar to this value: e.g. 58.29% (22), 59.29% (23), 76.8% (24), 86.61% (25) and 88.58% (26).

### Evaluation of larvicidal activity of clove EO and eugenol

Obtained LC_50_ of clove EO and eugenol against *An. stephensi* were 57 and 93ppm, respectively. LC_90_ values were 86 and 158ppm, respectively. There are many reports about larvicidal activities of other EOs against *An. stephensi*. For example, larvicidal activities (LC_50_) of some essential oils such as *Artemisia dracunculus* (11.36ppm), *Anethum graveolens* (38.80ppm) and *Kelussia odoratissima* (4.77ppm) were evaluated (27–29).

Determined LC_50_ of clove EO in this research is lower than many reports against *An. stephensi* For instance, *Lawsonia inermis* (69.40ppm) (30), *Cionura erecta* (77.30ppm) (31) and *Cupressus arizonica* (79.30ppm) (32), *Zhumeria majdae* (61.34ppm) (33). However, calculated LC_50_ in some other reports is lower than our reported LC_50_, for instance, *Bunium persicum* (27.72ppm) (34), *Tanacetum persicum* and *Achillea kellalensis* (48.64 and 35.42ppm respectively) (35), *Satureja bachtiarica* (24.27ppm) (36) and *Citrus aurantium* (31.20ppm) (37).

Larvicidal activity of either of clove EO and eugenol, against other species of mosquito, has already been reported. *Ae. aegypti* is shown to be more susceptible than *Cx. quinquefasciatus* when using clove EO (i.e. LC_50_: 92.56 vs. 124.42ppm) (13). Larvicidal activity of synthetic derivatives of eugenol against *Ae. aegypti* has been shown (LC_50_∼ 62.3ppm or higher) (14).

Recently, many reports have been released about comparison of larvicidal activity of EOs with their major components. However, there is no conclusion so far whether EOs outperforms or their major constituent in terms of larvicidal efficacy.

LC_50_ in *Allium tuberosum* EO against *Aedes albopictus* was found to be 17.9ppm, lower than that of its two major components, allyl methyl trisulfide and dimethyl trisulfide, with LC_50_ of 27.5 and 36.4ppm, respectively (38). LC_50_ of *Ruta chalepensis* EO has been evaluated against *Anopheles quadrimaculatus* (14.9 ppm) and *Aedes aegypti* (22.2ppm). While, 2-undecanone, its major component, showed similar LC_50_ to that of total EO against *An. quadrimaculatus* (14.2ppm), significantly lower values against *Ae. aegypti* (14.37ppm) were obtained (21).

LC_50_ of EO of *Allium macrostemon* (72.86 ppm) was better than a major constituent, methyl propyl disulfide (86.16ppm), while its efficacy was lower than the other major component, dimethyl trisulfide (36.36ppm), against *Ae. albopictus* (40).

Synergistic effects of constituents of some EOs are nowadays well-known when they are used as anti-fungal or anti-bacterial agents (39, 40). Our findings in this research and our previous study also show that *An. stephensi* is more susceptible to the clove EO or EO of *Kelussia odoratissima* (with LC_50_ of 57.49 and 4.77ppm, respectively), compared with their major constituents, eugenol (93.14ppm) and Z-ligustilide (8.73ppm), respectively (28). A type of synergism may have occurred in larvicidal activity of the EOs too.

EOs are mixtures of many constituents such as flavonoids, alkaloids, and monoterpenes (41, 42). Modes of action of mentioned constituents are different e.g. main site action of flavonoids is acetylcholinesterase (43), while alkaloids and monoterpenes target Na-K-ATPase or Na^+^ and K^+^ channels (44–46). This could be the main reason for occurring synergism in larvicidal activity in our study. Having mentioned that resistance against larvicides is mostly observed when a single active agent is used compared with those having multi components (47–49).

## Conclusion

Use of clove EO as a green larvicide against *An. stephensi* is preferred compared with its major constituent (Eugenol). Considering the fact that the EO is a lot cheaper than eugenol and is composed of several components, thus, has lesser chance of occurring resistance, the whole EO may be suggested as a proper larvicide.
